# Endotoxin induced peritonitis elicits monocyte immigration into the lung: implications on alveolar space inflammatory responsiveness

**DOI:** 10.1186/1465-9921-7-30

**Published:** 2006-02-18

**Authors:** Mirko Steinmüller, Mrigank Srivastava, William A Kuziel, John W Christman, Werner Seeger, Tobias Welte, Jürgen Lohmeyer, Ulrich A Maus

**Affiliations:** 1 Department of Internal Medicine, Division of Pulmonary and Critical Care Medicine and Infectious Diseases, Justus-Liebig-University, Giessen, Germany; 2 Protein Design Labs, Inc. Fremont, CA, USA; 3 University of Illinois at Chicago, IL, USA; 4 Department of Pulmonary Medicine, Laboratory for Experimental Lung Research, Hannover School of Medicine, Hannover, Germany

## Abstract

**Background:**

Acute peritonitis developing in response to gram-negative bacterial infection is known to act as a trigger for the development of acute lung injury which is often complicated by the development of nosocomial pneumonia. We hypothesized that endotoxin-induced peritonitis provokes recruitment of monocytes into the lungs, which amplifies lung inflammatory responses to a second hit intra-alveolar challenge with endotoxin.

**Methods:**

Serum and lavage cytokines as well as bronchoalveolar lavage fluid cells were analyzed at different time points after intraperitoneal or intratracheal application of LPS.

**Results:**

We observed that mice challenged with intraperitoneal endotoxin developed rapidly increasing serum and bronchoalveolar lavage fluid (BALF) cytokine and chemokine levels (TNFα, MIP-2, CCL2) and a nearly two-fold expansion of the alveolar macrophage population by 96 h, but this was not associated with the development of neutrophilic alveolitis. In contrast, expansion of the alveolar macrophage pool was not observed in CCR2-deficient mice and in wild-type mice systemically pretreated with the anti-CD18 antibody GAME-46. An intentional two-fold expansion of alveolar macrophage numbers by intratracheal CCL2 following intraperitoneal endotoxin did not exacerbate the development of acute lung inflammation in response to intratracheal endotoxin compared to mice challenged only with intratracheal endotoxin.

**Conclusion:**

These data, taken together, show that intraperitoneal endotoxin triggers a CCR2-dependent de novo recruitment of monocytes into the lungs of mice but this does not result in an accentuation of neutrophilic lung inflammation. This finding represents a previously unrecognized novel inflammatory component of lung inflammation that results from endotoxin-induced peritonitis.

## Background

Overwhelming innate immune responses to systemic inflammation contribute to the clinical manifestation of sepsis and septic shock. Acute respiratory distress syndrome (ARDS) and multiorgan failure are frequent complications of severe sepsis that contribute to the morbidity and mortality of this critical illness [1]. The molecular events eliciting sepsis-related complications such as acute lung injury have only been partially defined. For example, experimental animal models of endotoxin (lipopolysaccharide, LPS) induced acute peritonitis have characterized the kinetics of intraperitoneal LPS resorption and its rapid appearance within the vascular compartment within minutes to hours [2]. In human studies, patients who develop ARDS have highly elevated serum and alveolar cytokine and chemokine levels that are associated with a massive accumulation of neutrophils within the lungs, expanded alveolar macrophage populations and increased lung injury scores [3,4]. Key chemokines involved in this process of lung leukocyte invasion include the main monocyte chemoattractant CCL2 and the neutrophil chemoattractants MIP-2, KC and MIP-1α [4,5].

Although expansion of alveolar macrophage populations in septic ARDS patients is a well described phenomenon, the underlying molecular events shaping this leukocytic response are not known. In particular, it is unclear whether such expanded alveolar macrophage pools in septic ARDS patients are functionally relevant in terms of aggravating lung inflammatory responses to secondary pneumonia, which complicate the later phase of septic ARDS. Previously published studies employing a two hit model of initial intraperitoneal LPS challenge to trigger sepsis-induced remote lung inflammatory responses followed by a secondary intratracheal LPS challenge to mimic the additional development of pneumonia have not addressed changes in alveolar macrophage pool sizes and related functional consequences [2]. Clearly, both newly recruited alveolar monocytes and resident macrophages are potentially involved in the overall lung inflammatory response. We have recently demonstrated alveolar accumulations of CD14-positive mononuclear phagocytes and elevated BAL fluid CCL2 levels together with an expanded alveolar macrophage pool correlating with increased lung injury scores in patients with sepsis related ARDS [4]. In addition, we recently showed in an animal model that intratracheal application of CCL2 plus LPS synergistically induced acute lung inflammation, indicating that monocytes that are recruited into the lungs of mice may both expand the alveolar macrophage pool and aggravate LPS-induced neutrophilic alveolitis and lung injury in mice [6]. Thus, an expansion of alveolar macrophage pool sizes may be observed in both septic ARDS patients and in experimental animal models with ARDS-like inflammatory phenotypes [4,6], and expanded alveolar macrophage pools may be involved in remote lung injury developing in response to systemic inflammation [4,5]. Against this background, we hypothesized that LPS-induced peritonitis effects alveolar macrophage pool sizes by provoking the recruitment of circulating monocytes into the lungs, thereby amplifying lung inflammatory responses to a second hit intra-alveolar endotoxin challenge. We found that LPS-induced peritonitis was sufficient to elicit a robust twofold expansion of the alveolar macrophage pool without generating neutrophilic alveolitis. Using a two hit model of initial intraperitoneal plus intratracheal LPS application to mimic peritonitis-induced lung inflammation that is complicated by Gram-negative pneumonia, we found-contrarily to our initial hypothesis- that the observed alveolar macrophage expansion did not result in an aggravation of the overall lung inflammatory response to alveolar LPS challenge.

## Materials and methods

### Animals

BALB/c female mice (18–22 g) were purchased from Charles River (Sulzfeld, Germany). CCR2-deficient mice were generated on a mixed C57BL/6 × 129/Ola genetic background by targeted disruption of the CCR2 gene, as described previously [7]. The disrupted gene was backcrossed for six generations to wild-type BALB/c mice. Parent and offspring CCR2^-/- ^mice on the BALB/c background were bred under specific pathogen free (SPF) conditions. Animals 8–12 weeks old were used for the described experiments. Each treatment group consisted of at least 5 mice, unless indicated otherwise. This animal study was approved by the local government committee.

### Reagents

Lipopolysaccharide (*E. coli*, serotype O111:B4) was purchased from Sigma (Deisenhofen, Germany). Murine recombinant CCL2 (JE/MCP-1) was purchased from Peprotech (Tebu, Offenbach, Germany). Function-blocking rat antimurine CD18 antibody (clone GAME-46) was purchased from BD Biosciences (Heidelberg, Germany). All reagents and antibodies were ascertained to be endotoxin-free by Limulus amoebocyte lysate (LAL) assay (Chromogenix, Mölndal, Sweden).

### Treatment protocols

Intratracheal applications of the various inflammatory stimuli and recombinant proteins was done essentially as described elsewhere [5,6,8-11]. Briefly, tracheas of anaesthetized mice were surgically exposed, and an Abbocath catheter (Abbot, Wiesbaden, Germany) was inserted into the trachea. Subsequently, indicated concentrations of LPS or recombinant proteins (dissolved in total volumes of approximately 80 μl saline/0.1% endotoxin-free human serum albumin) were slowly instilled under stereomicroscopic control (Leica MS5, Wetzlar, Germany). Subsequently, the skin was sutured and mice were allowed to recover with free access to food and water.

Wild-type and CCR2-deficient mice (where indicated) were sedated with ketamine and received intraperitoneal (IP) injections of sterile LPS (50 μg/mouse) for various time intervals (0 h, 3 h, 6 h, 24 h, 48 h, 72 h, 96 h, 120 h, 144 h). In a second set of experiments, mice received initial intraperitoneal LPS applications, and at various time points thereafter, additional intratracheal (IT) applications of LPS (1 μg/mouse) to mimic the frequently observed clinical complication of pneumonia developing subsequent to the onset of septic ARDS that is associated with peritonitis. In a third set of experiments, mice received initial intraperitoneal LPS applications and 48 h later, received a single intratracheal application of recombinant CCL2 (50 μg/mouse) to further increase alveolar monocyte accumulations within the lung. At another 48 h post CCL2 application (i.e., 96 h after initial IP LPS application), mice were challenged intratracheally with LPS (1 μg/mouse) for 24 h. In a fourth set of experiments, mice received intratracheal applications of both CCL2 (50 μg/mouse) plus LPS (1 μg/mouse), according to recently published protocols [6,8,9]. The dose of 1 μg LPS/mouse was chosen to induce an easily detectable neutrophilic alveolitis in mice that also allows monitoring of changes in magnitudes and durations of the neutrophilic responses depending on the various experimental settings [11]. On the other hand, to elicit a robust monocytic response, we followed recently published experimental protocols and challenged mice intratracheally with 50 μg CCL2/mouse (where indicated). This strong CCL2 challenge has been described recently to establish a potent CCL2 chemokine gradient and subsequent selective monocyte recruitment towards the alveolar compartment [6,8,9]. For inhibition experiments, mice received intravenous injections of function-blocking anti-CD18 antibody GAME-46 (100 μg/mouse) 15 min prior to IP LPS application and every 24 h post IP LPS application. In initial experiments, repeated intraperitoneal GAME-46 antibody injections did not show any overt side effects and were well tolerated by the mice. Overall mortalities ranged below 10%.

### Collection and analysis of blood samples and bronchoalveolar lavage

Mice were sacrificed and blood sample and bronchoalveolar lavage collection was done as recently outlined in detail [6,8-10]. Briefly, mice were exposed to an overdose of isofluoran (Abbott, Wiesbaden, Germany) and blood was collected from the inferior vena cava. The bronchoalveolar lavage (BAL) fluid was obtained by cannulating the trachea with a shortened 21 G needle attached to a 1-ml insulin syringe, followed by repeated intratracheal instillations of 0.5 ml aliquots of PBS (pH 7,2, supplemented with 2 mM EDTA). Quantification of TNFα, MIP-2 and CCL2 proteins in BAL fluid and serum samples was performed using commercially available ELISA kits (lower detection limits, 2 pg/ml), as recommended by the manufacturer (R&D Systems, Wiesbaden, Germany). BAL cells were counted with a hemocytometer and quantitation of alveolar macrophages, alveolar recruited monocytes and neutrophils was done on differential cell counts of Pappenheim-stained cytocentrifuge preparations using overall morphological criteria, including differences in cell size and shape of nuclei and subsequent multiplication of those values with the respective total BAL cell counts, as recently described [10,11].

### In vivo lung permeability assay

For evaluation of IP LPS-induced lung permeability, mice received an intravenous injection of FITC-labeled human albumin (1 mg/mouse in 100 μl PBS; Sigma, Deisenhofen, Germany) 1 hour before death, as recently described [5,9]. Undiluted BAL fluid samples and serum samples (diluted 1:10 and 1:100 in PBS, pH 7.4) were placed in a 96-well microtiter plate and fluorescence intensities were measured using a fluorescence spectrometer (Bio-Tek FL 880 microplate fluorescence reader) operating at 488 nm absorbance and 525 ± 20 nm emission wavelengths, respectively. The lung permeability index is defined as the ratio of fluorescence signals of undiluted BAL fluid samples to fluorescence signals of 1:10 diluted serum samples.

### Statistics

The study data are expressed as mean ± SEM. Significant differences between controls and treatment groups of serum and BAL fluid cytokine levels were calculated by one-factor ANOVA with posthoc tests by Dunnet. Significant differences in numbers of alveolar macrophages and PMN were calculated by two-way ANOVA with post-hoc tests using the Dunnet procedure. Differences were assumed to be significant when *P *values were < 0.01.

## Results and discussion

**Figure 1:**
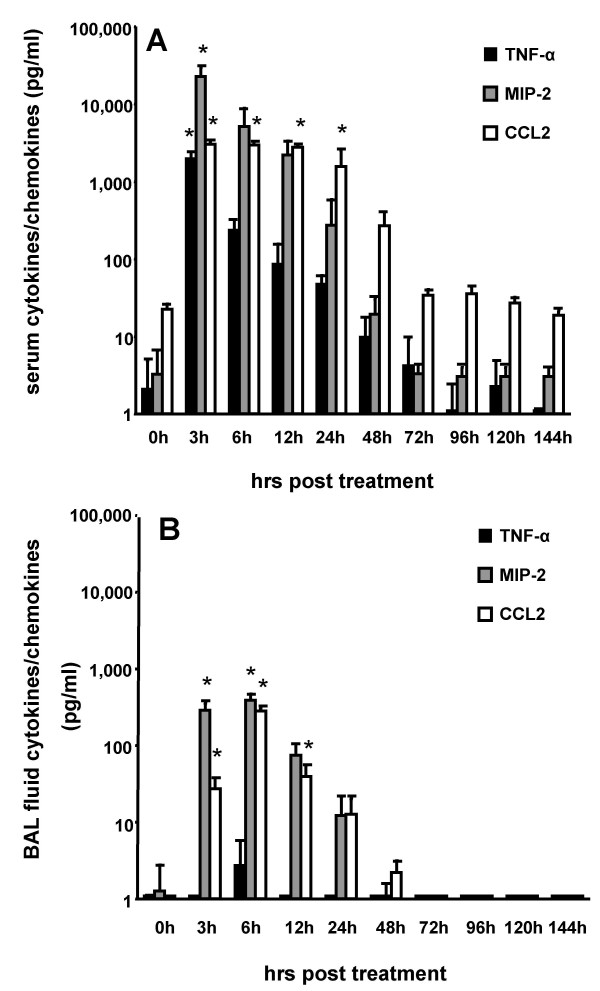
**Increased serum and bronchoalveolar lavage fluid TNFα, MIP-2 and CCL2 levels in mice in response to intraperitoneal endotoxin application**. Mice were either left untreated (0 h time points) or received a single intraperitoneal endotoxin application (50 μg/mouse). At various time points thereafter, mice were sacrificed and serum (A) and BAL fluid (B) TNFα (black bars), MIP-2 (grey bars) and CCL2 (white bars) levels were quantified by ELISA. The values are shown as mean ± SEM of 4–6 independent experiments. * indicates p < 0.01 versus respective control (0 h).

To evaluate the effect of systemic inflammation induced by intraperitoneal LPS application on remote lung inflammatory responses, mice were challenged with a single intraperitoneal LPS injection (50 μg/mouse) and changes in both serum and bronchoalveolar lavage fluid cytokine/chemokine profiles as well as BAL fluid cellular constituents were monitored at various time points thereafter. As shown in Fig. 1A, serum TNFα, MIP-2 and CCL2 levels dramatically (> 100-fold) increased in response to intraperitoneal application of LPS, peaking at 3 h and 6 h and returning to near baseline levels by 48 h after treatment, which is in agreement with previous reports [2,11]. At the same time, MIP-2 and CCL2 but not TNFα levels were also significantly increased in the BAL fluid compared to untreated controls, albeit at much lower levels (Fig. 1B). To evaluate, whether this increase in BAL fluid cytokines/chemokines was due to increased lung vascular leakage in response to intraperitoneal LPS treatment, mice received FITC-labeled albumin shortly before intraperitoneal LPS application, and FITC-albumin leakage into the alveolar air space was monitored at 3 h and 6 h post-treatment. However, we did not observe increased lung permeability in the IP LPS treated mice, which indicates that cytokine/chemokine leakage from the vascular to the alveolar compartment does not account for the elevated concentration in BAL fluid in our model (IP LPS (3 h), 0.10 ± 0.02 AU, IP LPS (6 h) 0.08 ± 0.01 AU, control, 0.12 ± 0.03 AU). This observation is different from that of Qu and coworkers who reported an induction of lung permeability in a rat model of induced endotoxin tolerance provoked by repeated intraperitoneal LPS applications. Qu et al. employed high-dose LPS injections amounting to 6 mg/kg body weight, corresponding to ~1,200 μg LPS/rat, whereas we used a single medium dose LPS application amounting to 2,5 mg/kg body weight, corresponding to ~50 μg LPS/mouse [12]. Differences in lung permeability changes observed between these two studies are most probably due to differences in experimental settings and animal models employed. In addition, since systemic LPS is known to rapidly sequester circulating leukocytes within lung capillaries and interstitium, such leukocytes likely prime alveolar epithelial cells to release chemokines and other proteins into the alveolar air space in a polarized manner, which may represent an alternative possibility to increase BAL fluid protein contents in the absence of increased lung permeability [13]. The latter scenario is supported by the observation that systemic anti-CD18 antibody application to block leukocyte-endothelial cell interactions was effective in lowering BAL fluid proinflammatory cytokine levels in acute lung inflammation [5].

**Figure 2:**
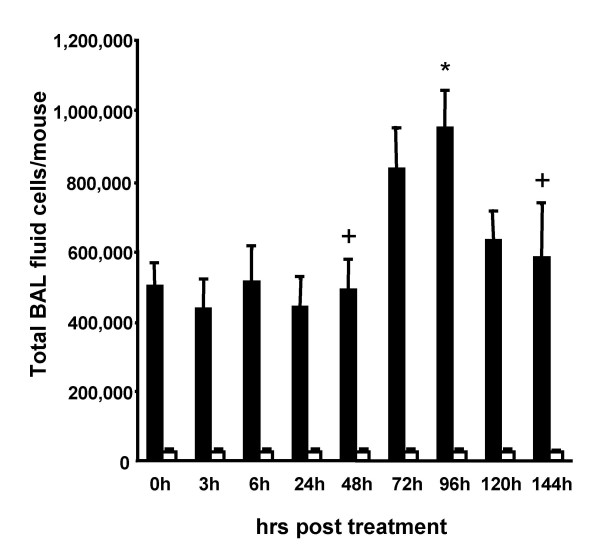
**Intraperitoneal endotoxin application elicits alveolar macrophage expansion in mice**. Mice were either left untreated (0 h time point) or were challenged intraperitoneally with a single dose of endotoxin (50 μg/mouse). At various time points post treatment, mice were sacrificed and subjected to bronchoalveolar lavage for determination of alveolar macrophage (black bars) and neutrophil (white bars) numbers, as indicated. Values are given as mean ± SEM of six mice per treatment group (96 h time point, n = 15). * indicates p < 0.01 versus control (0 h), + indicates p < 0.01 versus 96 h time point.

**Figure 3:**
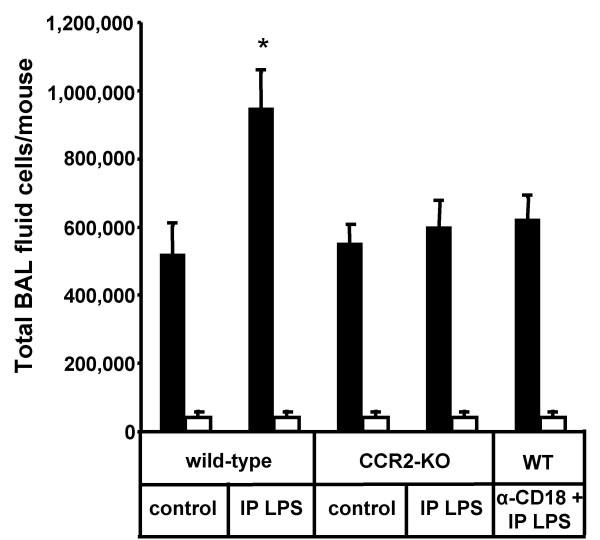
**Alveolar macrophage expansion elicited in endotoxin-induced peritonitis is CCR2-dependent**. Wild-type mice or CCR2-deficient mice were either left untreated or received a single intraperitoneal LPS application (50 μg/mouse) or wild-type mice were pretreated with function-blocking anti-CD18 antibodies prior to and every 24 h post intraperitoneal LPS application, as indicated. At 96 h post-treatment, mice were sacrificed and alveolar macrophages (black bars) and neutrophils (white bars) contained in bronchoalveolar lavage were quantified. Values are given as mean ± SEM of five mice per treatment group. * indicates p < 0.01 compared to all other treatment groups.

Evaluation of BAL fluid cellular constituents revealed that intraperitoneal LPS application provoked a delayed and robust two-fold expansion of the alveolar macrophage population by 96 h post-treatment (Fig. 2). However, under the chosen experimental conditions (50 μg LPS/mouse intraperitoneally), we observed no neutrophil recruitment into the alveolar compartment. In an effort to dissect the molecular pathways mediating this novel and unexpected lung inflammatory response to intraperitoneal LPS application, mice lacking the CCL2 receptor, CCR2 were challenged intraperitoneally with LPS. In contrast to wild-type mice, CCR2-deficient mice did not respond with an alveolar macrophage expansion upon intraperitoneal LPS application, which indicates that lung inflammatory monocyte trafficking observed in IP LPS challenged wild-type mice is dependent on CCL2 interacting with its receptor, CCR2 as underlying mechanism (Fig. 3) [6,9]. Moreover, since inflammatory monocyte trafficking to the lung is known to depend on engagement of β2 integrins [9], wild-type mice received function-blocking anti-CD18 antibodies to block β2 integrin-dependent molecular pathways prior to intraperitoneal LPS application. Importantly, this experimental manoeuvre completely inhibited the alveolar macrophage expansion in response to intraperitoneal LPS (Fig. 3). Although alveolar macrophage expansion has been described in septic ARDS patients [3,4], the currently presented data are novel and expand previous observations by demonstrating that the process of intra-alveolar macrophage expansion in response to remote (ie, intraperitoneal) LPS application involves a β2 integrin-dependent and CCL2-CCR2-mediated de novo recruitment of peripheral blood monocytes into the alveolar air space. In addition or alternatively to putative alveolar macrophage proliferation discussed for inflamed lungs [14], de novo recruitment of monocytes to the alveolar space may thus represent a robust mechanism to increase the alveolar macrophage pool.

**Figure 4:**
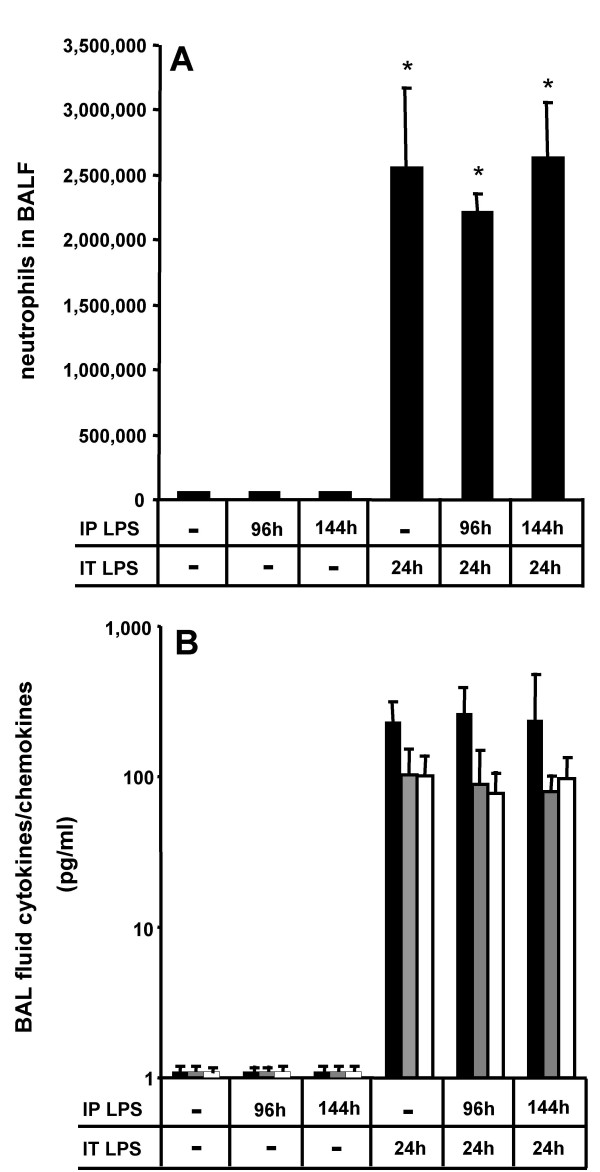
**Alveolar macrophage expansion elicited by intraperitoneal LPS application does not enhance neutrophilic alveolitis and cytokine release in response to subsequent intratracheal LPS application**. Mice were either left untreated or received an intraperitoneal LPS application (50 μg/mouse) for either 96 h or 144 h or intratracheal LPS alone (1 μg/mouse) for 24 h or combinations of intraperitoneal LPS followed by intratracheal LPS application, as indicated. Subsequently, bronchoalveolar lavage fluid neutrophil numbers (A) and BAL fluid (B) TNFα (black bars), MIP-2 (grey bars) and CCL2 (white bars) levels were quantified by ELISA. Values are given as mean ± SEM of at least five mice per treatment group. * indicates p < 0.01 versus control.

We and others recently demonstrated that alveolar macrophages are essential to the initiation of LPS-induced acute lung inflammation [10,15]. Liposomal clodronate-induced alveolar macrophage depletion prior to intratracheal LPS application largely abrogated LPS-induced cytokine liberation and neutrophilic alveolitis in mice [9,13]. Against this background, we further hypothesized that increased alveolar macrophage numbers observed in the lungs of mice pretreated intraperitoneally with LPS strongly amplify the alveolar inflammatory response to an additional alveolar LPS challenge, reflecting the frequently observed clinical complication of developing pneumonia in septic ARDS patients. Again, intraperitoneal LPS application alone did not provoke any substantial intra-alveolar neutrophil accumulation, irrespective of the time point investigated, whereas intratracheal LPS application alone induced a strong alveolar neutrophil recruitment by 24 h post-treatment (Fig. 4). However, when mice were challenged intratracheally with LPS at the time point where maximum alveolar macrophage accumulation triggered by intraperitoneal LPS application was observed (ie, at 96 h post intraperitoneal LPS treatment, see Fig. 2), the developing neutrophilic alveolitis and associated BAL fluid cytokine/chemokine levels was in the same order of magnitude as observed in those mice receiving only intratracheal LPS applications for 24 h in the absence of intraperitoneal LPS pre-challenge (Fig. 4A, B). Similar observations regarding the extent of neutrophilic alveolitis and BAL fluid cytokine/chemokine levels were made when mice received intratracheal LPS applications at 144 h post intraperitoneal LPS challenge, when alveolar macrophage numbers had already returned to near baseline values (Fig. 4, and see Fig. 2 for comparison). Collectively, the data show that robust increases in alveolar macrophage numbers developing in mice at 96 h post systemic LPS application did not increase the release of proinflammatory mediators within the alveolar air space nor aggravated the neutrophilic alveolitis provoked by a secondary intra-alveolar LPS challenge.

**Figure 5:**
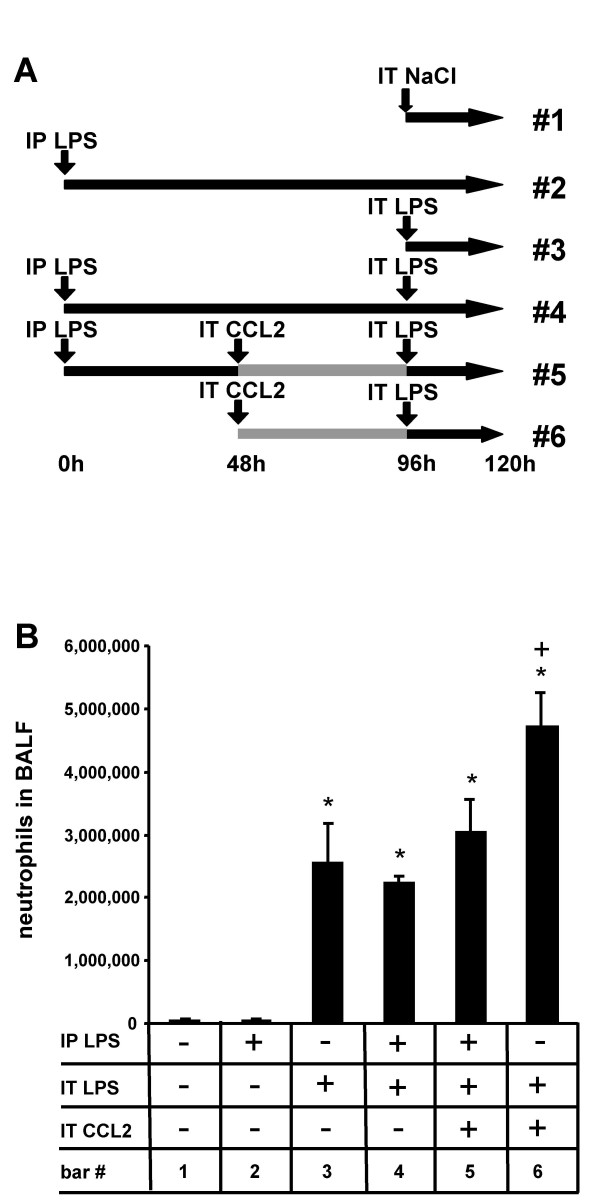
**Monocytes recruited into the lungs in response to intraperitoneal LPS do not enhance but rather decrease neutrophilic alveolitis in mice challenged with CCL2 plus LPS**. (A) Treatment groups of experiments shown in (B). Mice were either left untreated (bar 1) or received LPS intraperitoneally for 96 h (bar 2) or received intratracheal LPS applications alone for 24 h (bar 3) (1 μg/mouse). In addition, groups of mice received either combined intraperitoneal LPS applications (50 μg/mouse) and 96 h later, an intratracheal LPS application for 24 h (1 μg/mouse) (bar 4) or combined intratracheal applications of CCL2 for 48 h (50 μg/mouse) plus LPS for 24 h (1 μg/mouse) (bar 6) or were pretreated with intraperitoneal LPS for 48 h, followed by instillation of CCL2 (50 μg/mouse) for another 48 h followed by an intratracheal LPS application for 24 h (bar 5). Subsequently, mice were sacrificed and total numbers of alveolar recruited neutrophils were calculated. Values are given as mean ± SEM of at least five mice per treatment group. * indicates p < 0.01 versus control, + indicates p < 0.01 compared to all other treatment groups.

Intra-alveolar deposition of high doses of recombinant CCL2 (50 μg/mouse) employed to trigger a maximal cellular response has recently been shown to recruit substantial numbers of circulating monocytes into the lungs of mice [8,9]. In the current study, we used this approach to further expand the alveolar macrophage pool over that occurring in response to intraperitoneal LPS application alone (data not shown) and questioned, whether the cumulative alveolar macrophage expansion resulting from both initial intraperitoneal LPS plus additional intratracheal CCL2 instillation would ultimately increase the alveolar neutrophilic response upon subsequent intratracheal LPS application. As shown in Fig. 5, mice treated with both intraperitoneal LPS plus intratracheal CCL2 applications developed a neutrophilic alveolitis upon secondary intratracheal LPS similar to that observed in mice pretreated with intraperitoneal LPS plus secondary intratracheal LPS application 96 h later (Fig. 5, bar 5 vs. 4). In contrast, when untreated mice received an intratracheal CCL2 instillation for 48 h followed by an intratracheal LPS application for another 24 h, neutrophilic alveolitis was significantly increased, consistent with recently published reports (bar 6) [5,6]. Of note, the differences in developing neutrophilic alveolitis were not due to different numbers of newly recruited alveolar monocytes between these two treatment groups (IP LPS plus IT CCL2 plus IT LPS (bar 5), 191,500 ± 20,000 monocytes versus IT CCL2 plus IT LPS (bar 6), 189,500 ± 20,000 monocytes).

Thus, in sharp contrast to what we initially expected, these data lend support to the concept that monocytes recruited from inflamed vascular compartments into the lungs apparently do not aggravate neutrophilic alveolitis in response to secondary intra-alveolar LPS challenge, whereas alveolar monocytes recruited from non-inflamed vascular compartments significantly contribute to neutrophilic alveolitis upon intratracheal LPS application, the latter being consistent with recent reports [5,6].

One possible explanation why the increased lung monocyte traffic leading to an expansion of the alveolar macrophage pool in response to intraperitoneal LPS did not aggravate the neutrophilic alveolitis upon subsequent intratracheal LPS application may involve changes in TLR expression profiles on circulating monocytes pre-exposed to systemic LPS (absorbed from the peritoneal cavity) prior to being recruited into the lungs [16,17]. Such monocytes might exhibit a reduced potential to mount proinflammatory responses within the alveolar air space upon secondary challenge with locally applied LPS. Such attenuation of alveolar inflammatory responses might affect the lung host defense under conditions of systemic inflammation. This hypothesis of a dysregulated TLR gene expression pattern in LPS "pre-exposed" circulating monocytes is currently being addressed in our lab in more detail.

## Conclusion

Together, the presented data for the first time show that intraperitoneal LPS application in mice elicits a remote lung inflammatory response reflected by a CCR2-dependent and β2 integrin mediated robust expansion of the alveolar macrophage pool. However, no evidence was found to demonstrate that the observed monocyte immigration and resulting alveolar macrophage expansion subsequent to systemic inflammation leads to an aggravated neutrophilic alveolitis developing in response to a "second hit" alveolar LPS application. This finding is opposite to the enhanced neutrophilic alveolitis observed in mice where monocytes were recruited from a non-inflamed vascular compartment to the lungs (ie, in response to intratracheal CCL2 plus LPS) to expand alveolar macrophage numbers. These data may contribute to a better understanding of the inflammatory capacity of mononuclear phagocytes in the lungs of septic ARDS patients.

## Competing interests

The author(s) declare that they have no competing interests.

## Authors' contributions

M Steinmüller carried out the experimental studies and drafted the manuscript. M Srivastava helped with the experimental work. WA Kuziel provided genetically modified mice and participated in the design of the experiments. JW Christman, W Seeger and T Welte participated in the experimental design. J Lohmeyer and UA Maus initiated the study, supervised the experimental work and participated in the manuscript preparation. All authors read and approved the final manuscript.
